# Innovative Honey-Based Product and Its Beneficial Effects Measured by Modern Biophysical and Imaging Skin Techniques

**DOI:** 10.3390/ph17121709

**Published:** 2024-12-18

**Authors:** Grzegorz Suwiński, Izabela Nowak

**Affiliations:** 1 Department of Applied Chemistry, Faculty of Chemistry, Adam Mickiewicz University, Uniwersytetu Poznańskiego 8, 61-614 Poznań, Poland; 2 Research and Developement Department, Przedsiębiorstwo Farmaceutyczne Farmapol Sp. z o. o, Święty Wojciech 29, 61-749 Poznań, Poland

**Keywords:** formulation, honey, hydration, anti-wrinkle, TEWL, stickiness, skin

## Abstract

Background: Honey is widely recognized for its potential benefits in skincare, yet its incorporation into formulations is challenging due to its stickiness. This study aimed to evaluate the effects of hand creams with varying concentrations of multifloral honey (0%, 5%, 10%, and 15% *w*/*w*) on skin parameters and to assess their application characteristics. Methods: A total of 24 volunteers were divided into two groups, each receiving a blinded pair of creams (0%/10% or 5%/15% honey) to apply on their left and right hands. Instrumental methods (Corneometer^®^, Tewameter^®^, Cutometer^®^, Visioscan^®^, and Visioline^®^) were used to measure skin parameters at the baseline, 15 min after the first application, and after 4 weeks of regular use. Sensory characteristics were evaluated through participant questionnaires. Results: After 4 weeks, honey-infused creams showed notable improvements in skin moisturization (up to 29.7%), smoothness (up to 21.3%), wrinkle area reduction (up to 21.4%), and mean wrinkle depth reduction (up to 11.7%). Among these, the increases in moisturization and reductions in wrinkle depth were statistically significant compared to the placebo. The sensory evaluations revealed no significant differences between formulations, highlighting the vehicle’s effectiveness in minimizing the stickiness typically associated with honey. Conclusions: This study highlights the efficacy of honey-enriched hand creams in enhancing skin parameters over time while maintaining favorable sensory characteristics. These findings support the use of honey in dermatological formulations and provide insights into overcoming its formulation challenges.

## 1. Introduction

The global natural cosmetics market is experiencing rapid growth. According to forecasts, the market is projected to reach USD 48.4 billion in 2023 and is expected to further increase to USD 79.6 billion by 2033, with a compound annual growth rate of 5.1% [1]. Skincare products are estimated to constitute a significant portion of the cosmetic market, accounting for around 36% of its total value [2]. This attractive business sector must address evolving consumer demands, particularly in terms of product concept, packaging, effectiveness, trends in active substances, and formulation application parameters [3].

Despite their considerable marketing potential and relatively high efficacy, natural ingredients may pose challenges in maintaining the stability and sensory quality of the final cosmetic product. As exemplified in this paper, the formulation of cosmetic products with a high content of bee honey presents particular challenges. Honey consists of over 181 substances, many of which have positive effects on the skin. Notable among these substances are organic acids, flavonoids, proteins, ascorbic acid, carotenoids, glucose, and fructose [4]. While these carbohydrates often constitute the majority of the composition (approximately 70% *w*/*w*), their proportions can vary based on the origin of the honey [5]. Although the simple sugars in honey are potent humectants, they can also lead to undesirable stickiness after cosmetic application [6]. This stickiness is attributed to the low glass transition temperature, high viscosity, and hygroscopic nature of glucose and fructose [7,8]. When cosmetics with a high honey content are applied, water can evaporate, leaving honey residue on the stratum corneum. At a microscopic level, glucose and fructose do not solidify into a brittle state; instead, they become glassy, viscous, and sticky. This phenomenon is responsible for a sticky sensation that many consumers may find unpleasant [9]. However, this effect can be managed through various formulation methods, such as including emollients in anhydrous formulations to mitigate the stickiness caused by honey [10]. Nevertheless, reports occasionally mention the usage of popular cosmetic formulations with a constant water phase, including solutions, gels, and O/W (oil in water) emulsions with a high (>5–10% *w*/*w*) honey content.

Pavlačková and coworkers investigated the short-term effects of 5% and 10% (*w*/*w*) honey O/W emulsions. The moisturizing effect on the skin increased with higher concentrations of honey, although sensory analysis showed no significant differences between the formulations. The team also demonstrated the impact of honey type on skin parameters: floral honey, containing more reducing sugars, had a greater effect on skin hydration than forest honey, which is lower in these molecules [11]. Another study by Isla and colleagues examined formulations containing various types of honey at a level of 15% (*w*/*w*). Each formula exhibited high moisturization effects (approximately 50%), but only Tetragonula carbonaria honey demonstrated exceptional DPPH free radical (2,2-diphenyl-1-picryhydrazyl) sequestration ability (71% inhibition) [12].

In the literature, honey is a well-studied substance that has demonstrated multiple positive effects on the skin, particularly as a cosmetic ingredient and a wound dressing. In addition to its moisturizing properties, it exhibits potent antimicrobial and regenerative impacts due to its high osmolarity and enzyme content, particularly lysozyme and glucose oxidase [13]. The remarkable healing capabilities of honey, particularly in higher concentrations, encompass wound regeneration [14]. Beyond traditional medical applications, honey holds potential in facial care cosmetics, including wrinkle reduction, pore refinement, and sebum control [15]. This paper presents the application effects of short-term and long-term use, together with the questionnaire results, on skin biophysical parameters for O/W hand cream formulations with varying honey contents (0%, 5%, 10%, 15% *w*/*w*). One can expect that the ingredients of honey will use the osmotic power of sugar as well as its humectant properties to soften the skin; thus, the cosmetic properties will be enhanced. Our study has several strengths, i.e., this is the first study describing the dynamic trends of multiple skin barrier parameters, supporting the statement that honey controls skin hydration. Additionally, this study aims to identify the optimal concentration of honey for new products introduced to the market.

## 2. Results

**Figure 1:**
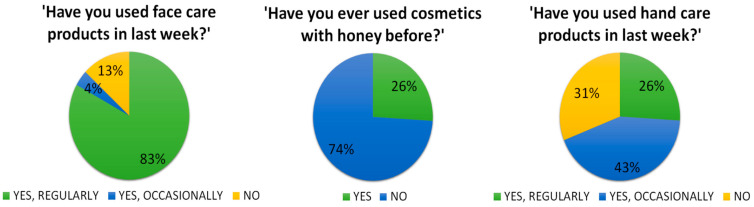
Questionnaire responses of the examined group.

Individual responses related to the use of cosmetics are depicted in Figure 1. Among the participants, 74% did not recognize honey as a component in cosmetics they previously used. A comparison of daily cosmetic care habits revealed greater interest in facial care (83% of respondents used it regularly) as opposed to hand care (26% of respondents used it regularly).

**Figure 2:**
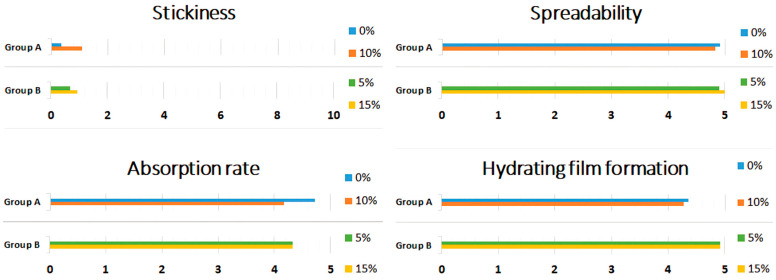
Honey cream sensations were determined employing response ratings (0–5), presented in tested pairs: Group A (0%, 10% *w*/*w* honey) and Group B (5%, 15% *w*/*w* honey).

The questionnaires did not indicate any statistically significant differences (*p* > 0.05) in the characteristics of hand cream pairs (left and right hand) within both Group A (0%/10% *w*/*w* honey) and Group B (5%/15% *w*/*w* honey). The evaluation of stickiness, spreadability, absorption rate, and the sensation of a hydrating film on the skin after cream usage is presented as group means in Figure 2.

**Figure 3:**
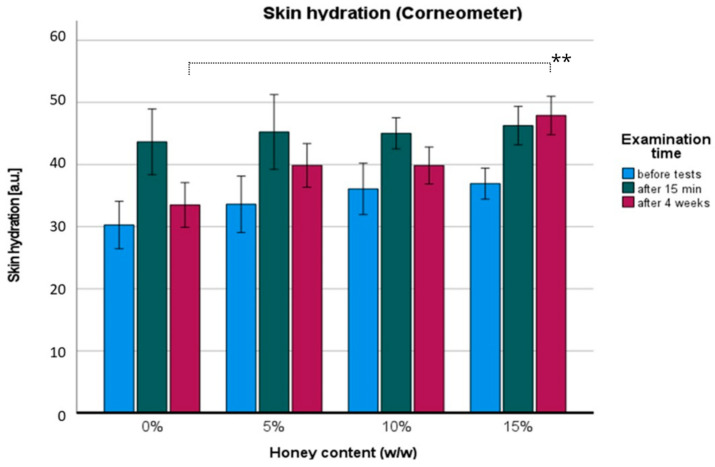
Skin hydration: before (blue), 15 min after first use (dark green), and after four weeks of regular use (maroon red) of hand honey creams. Significance level: ** *p* < 0.001.

Monitoring stratum corneum hydration, TEWL (transepidermal water loss), skin elasticity, and microrelief are used to evaluate skin function. A quantitative description of these parameters will facilitate a comprehensive evaluation of skin status. The skin hydration effects of the four tested formulations are presented in Figure 3. The ANOVA results showed statistically significant differences in terms of the honey percentage: F (3,66) = 7.34; *p* < 0.01; η = 0.4, in time of examination: F (2,66) = 56.04; *p* < 0.001; η = 0.84, and in percentage with time dependence: F (6,66) = 3.09; *p* < 0.01; η = 0.22. All groups had no statistical differences after 15 min of initial usage (*p* > 0.05). However, significant differences were observed between the placebo (0% *w*/*w* honey) and each other cream after four weeks of regular use, with the effect size correlating with the cream percentage (*w*/*w*): 5% (d = 0.72), 10% (d = 0.97), and 15% (d = 1.27). Post hoc analysis with Bonferroni correction revealed a significant hydration increase between the placebo and 15% *w*/*w* honey cream (*p* < 0.001). By comparing average values before and after four weeks, the highest skin hydration effect was exhibited by the 15% *w*/*w* honey cream (+29.7% a.u.) compared to the placebo (+10.6% a.u.).

**Figure 4:**
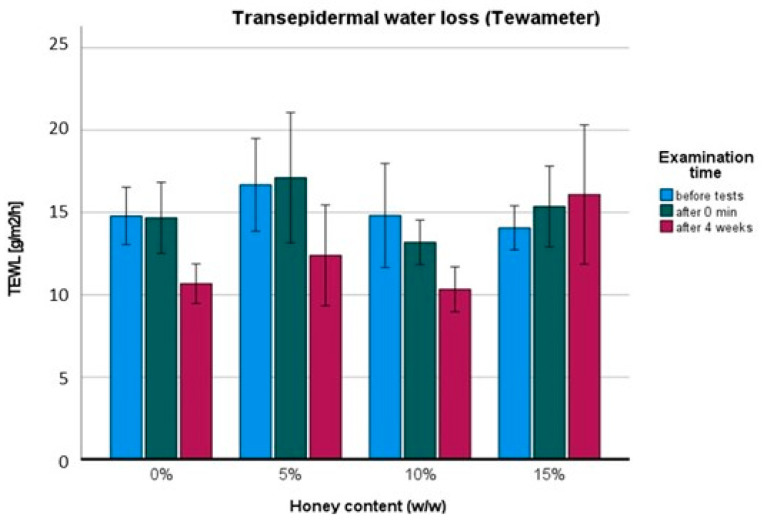
Transepidermal water loss: before (blue), 15 min after first use (dark green), and after four weeks of regular use (maroon red) of hand honey creams.

Skin barrier function, examined with the Tewameter probe, i.e., the measurement of the transepidermal water loss, is presented in Figure 4. The ANOVA showed statistically significant differences in terms of the honey percentage: F (3,66) = 3.04; *p* < 0.05; η = 0.22, and in the time of examination: F (2,66) = 6.58; *p* < 0.01; η = 0.37. Bonferroni correction revealed no differences between groups. After the first use, a single statistical difference was identified between the 5% *w*/*w* and 15% *w*/*w* groups t (11) = 2.67; *p* < 0.05. Four weeks of regular use of the hand creams generated a decrease in TEWL (*p* < 0.05), except for the 15% *w*/*w* honey cream, which had no statistically significant effect (*p* > 0.05). There was no difference between the placebo, 5% *w*/*w*, and 10% *w*/*w* honey creams (*p* > 0.05). However, the 15% *w*/*w* honey cream differed from the placebo (*p* < 0.05; d = 0.76), the 5% *w*/*w* honey cream (*p* < 0.05; d = 1.05), and the 10% [*w*/*w*] honey cream (*p* < 0.05; d = 0.78). The reduction in TEWL after four weeks was similar for the placebo (−27.8%) and the 5% honey cream (−25.7%).

**Figure 5:**
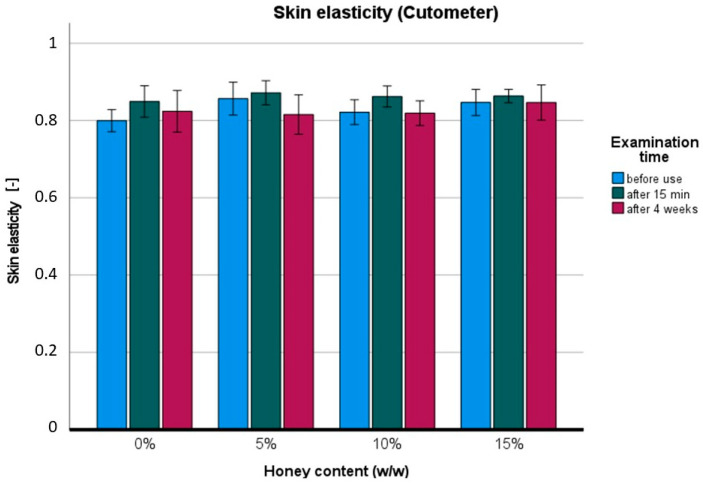
Skin elasticity: before (blue), 15 min after first use (dark green), and after four weeks of regular use (maroon red) of hand honey creams.

Skin elasticity, calculated using the Cutometer method, is visualized in Figure 5. The ANOVA showed statistically significant differences in terms of time: F (2,66) = 6.74; *p* < 0.01; η = 0.38. However, the *t*-tests did not reveal any statistically significant differences between the groups 15 min after the first use and after four weeks of regular use.

**Figure 6:**
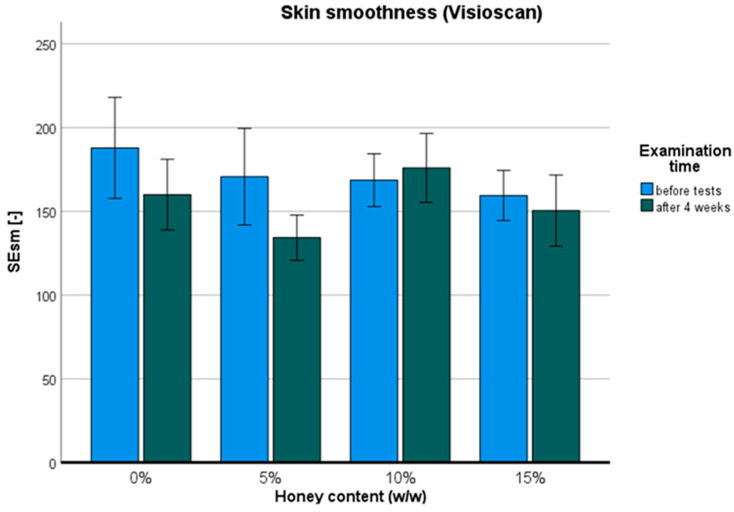
Skin smoothness: before (blue) and after four weeks of regular use (dark green) of hand honey creams.

**Figure 7:**
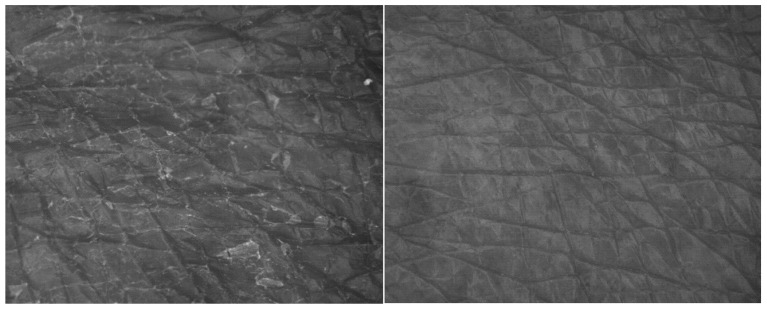
A single example of skin smoothness improvement by 43% presented with Visioscan VC 98 USB camera before treatment (**left**) and after four weeks of 5% (*w*/*w*) honey cream treatment (**right**).

The skin smoothness parameter was obtained using Visioscan (Figure 6). The ANOVA revealed a statistically significant difference over time: F (1,11) = 5.841; *p* < 0.05; η = 0.35, as well as with varying percentages of honey: F (3,33) = 2.923, *p* < 0.05, η = 0.210. There were no significant differences observed between groups before the test, but significance was identified with Student’s *t*-tests after four weeks between the 5% *w*/*w* and 10% *w*/*w* groups: t (11) = −4.732; *p* < 0.001, as well as between the 10% *w*/*w* and 15% *w*/*w* groups: t (11) = 2.453; *p* < 0.05. The only statistically significant effect of the treatment was observed in the 5% *w*/*w* group: t (11) = 2.816; *p* < 0.05. With the 5% *w*/*w* honey cream, skin smoothness improved by an average of 21.3% after 4 weeks of regular use. An example displaying a 43% improvement is depicted in Figure 7. Additionally, liquid water uptakes action on the surface of the stratum corneum to reduce light reflection and backscatter. This visible decrease in internal scattering is a result of hydration.

**Figure 8:**
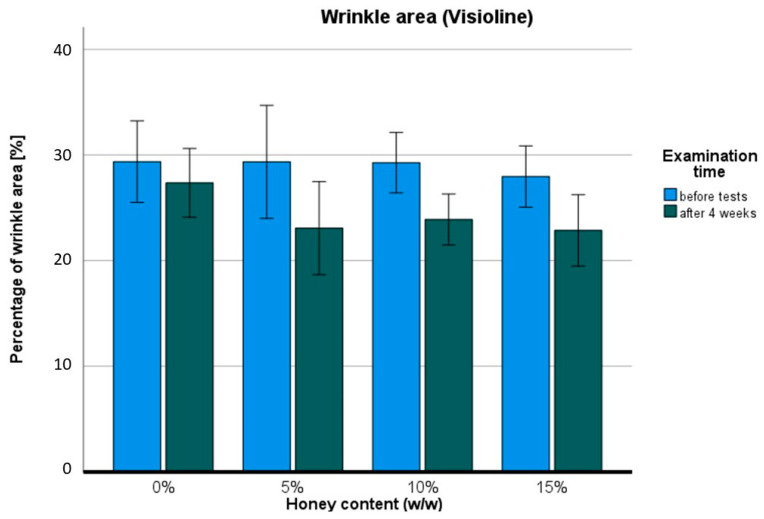
Wrinkle area: before (blue) and after four weeks of regular use (dark green) of hand honey creams.

The skin wrinkle area is shown in Figure 8. The ANOVA revealed statistically significant differences in terms of time: F (1,11) = 11.325; *p* < 0.01; η = 0.507. Slightly distinguishable statistical differences were observed after four weeks of use between the 0% *w*/*w* and 15% *w*/*w* groups t (11) = 2.175, *p* = 0.05. However, differences were found within individual groups before and after honey cream treatment, specifically in the 5% *w*/*w* group t (11) = 2.531; *p* < 0.05, the 10% *w*/*w* group t (11) = 3.193; *p* < 0.01, and the 15% *w*/*w* group t (11) = 2.596; *p* < 0.05. No statistical difference was observed in the placebo group. The skin wrinkle area was reduced by around 20% (−18.25% to −21.29%) after four weeks of using each honey cream. 

**Figure 9:**
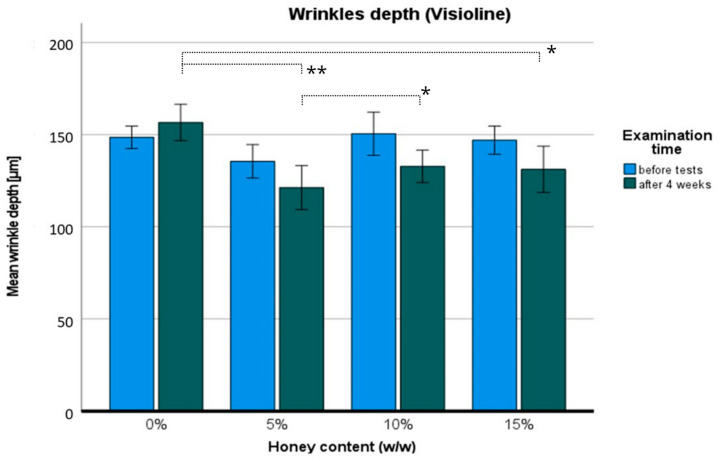
Wrinkles depth: before (blue) and after four weeks of regular use (dark green) of hand honey creams. Significance level: * *p* < 0.05; ** *p* < 0.001.

**Figure 10:**
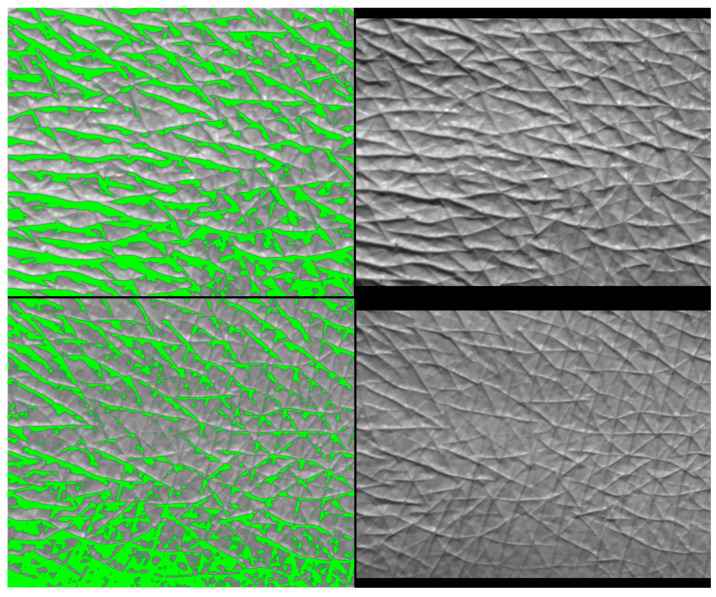
An example of mean wrinkle depth improvement by 22,7% with 10% honey cream care, visualized with Visioline VL 650–Quantridies by skin replica illumination, camera registration (right-side pictures), and computer analysis (left-side pictures). The upper pictures present the skin surface before treatment and the bottom pictures present the skin after 4 weeks of honey cream treatment.

The wrinkle depth of the hand skin is illustrated in Figure 9. The ANOVA revealed statistically significant differences in terms of the following variables: time F (1,11) = 13.116; *p* < 0.01; η = 0.544, in the percentage of honey: F (3,33) = 17.575; *p* < 0.001; η = 0.615, and the percentage with time dependence F (3,33) = 3.44; *p* < 0.05; η = 0.238. Post hoc analysis proved differences between the placebo and 5% *w*/*w* honey cream (*p* < 0.001), as well as the 15% *w*/*w* honey cream (*p* < 0.05) and between the 5% *w*/*w* and 10% *w*/*w* honey cream (*p* < 0.05). *t*-tests indicated differences in every group compared to the placebo: 5% *w*/*w*: t (11) = 6.266; *p* < 0.001, 10% *w*/*w*: t (11) = 3.778; *p* < 0.01, and 15% *w*/*w*: t (11) = 3.892; *p* < 0.01, respectively. When comparing individual groups before and after the honey treatment, statistical differences were found in the 10% *w*/*w* group t (11) = 2.917; *p* < 0.05 and the 15% *w*/*w* group t (11) = 2.456; *p* < 0.05. Wrinkle depth was reduced by around 10% (10.5–11.7%) with honey creams, while the placebo seemed to have the opposite effect (+5.4% wrinkle depth). An example of skin analysis with Visioline VL 650-Quantridies is presented in Figure 10.

## 3. Discussion

Honey is well known for its health benefits, both in food and cosmetics. According to a survey of 600 Polish respondents, 95.9% of the population included honey in their diet [16]. Honey is also a popular and commonly available cosmetic ingredient in the market. Its attractiveness in cosmetics was confirmed by approximately two-thirds of a group examined by Żak and Przybyłowski [3]. In a Polish study involving 487 people, 42.9% declared using cosmetics with honey in the past [17]. In our study, only 26% of participants declared usage of honey-infused cosmetics in the past, which could be attributed to the small study group. However, this lower result is desirable as it helps to reduce bias by minimizing preconceived notions about the tested cosmetics.

A study in France examined the use of different cosmetic categories. Among adult women, 77% used face moisturizers and 81% used hand moisturizers [18]. In our study, the results were quite similar but opposite in categories. More participants used face moisturizers (87%) compared to hand moisturizers (69%). This discrepancy could be attributed to different cosmetic usage habits among French and Polish consumers.

The sensory and textural parameters of cosmetic formulations are commonly analyzed using sensory panelists with a rating scale and instrumental analysis with a texturometer [19]. For this study, we chose a sensory panel to reveal the complex and subjective perceptions of consumers. The four formulations were assessed as similar, with no statistically significant differences in the evaluation of sensory parameters for each cream. This desirable outcome indicates that the chosen cream base effectively masked the honey content, particularly in terms of stickiness.

Honey’s composition, rich in sugars, is believed to act as a nourishing and moisturizing active substance. The presence of free hydroxyl groups in reducing sugars attracts water through hydrogen bonding, increasing the overall water content in the stratum corneum. However, the level of humectant moisturization dependent on honey percentage may be debatable. For instance, Jarząbek-Perz and coworkers did not find a statistically significant difference between the use of 10% and 30% (*w*/*w*) gluconolactone in a facial formulation [20]. Our study showed a growing moisturizing effect in a honey percentage-dependent manner after a four-week usage period. The higher the honey concentration, the greater the increase in skin moisture detected with prolonged cream use. This could be explained by sugar accumulation in the stratum corneum or by the nourishing effect of other honey components, such as polyphenols. In contrast, short-term use did not show any statistically significant moisturization differences between the groups. This is expected, as each formulation provided additional water, instantly correcting the skin’s condition.

Different results were obtained with the Tewameter apparatus. The TEWL parameter reflects the skin’s barrier function and is useful for measuring barrier creams, especially those containing ceramides, sebum, and the NMF (Natural Moisturizing Factor). Effective ceramide creams have been shown to reduce TEWL in both the short term (−22% reduction) and long term (−68% reduction) [21,22].

In our study, four weeks of regular use caused decreased transepidermal water loss for each group except for the 15% *w*/*w* honey cream, which showed no statistically significant difference before and after treatment. Similarly, no statistical difference was observed in each group 15 min after the first use. A reduction in TEWL in moisturizing O/W creams was not anticipated. However, the phenomenon observed with the 15% *w*/*w* honey cream is surprising. Typically, a high concentration of humectant increases skin hydration and reduces TEWL, but there are cases where these substances do not positively affect the improvement in the skin barrier [23]. An excessively high concentration of humectant could potentially act similarly to weak penetration enhancers, disrupting the lipid structure of the epidermis [24]. However, there are no scientific reports supporting these hypotheses with regard to honey. Further studies are required to understand this phenomenon.

Skin elasticity is closely related to the dermis layer of the skin, where collagen and elastic fibers are present. These flexible structures are synthesized by fibroblasts [25]. The mechanism of activating fiber synthesis or decreasing degradation is therefore related to pharmacodynamic actions rather than overall skin moisturization. It is challenging for scientists to develop a formulation that significantly affects skin elasticity, usually requiring pharmacologically active substances incorporated into the appropriate vehicle. For instance, G. Jeswani and S. Saraf prepared nanosized ethosomes with *Curcuma longa* L. extract. After two weeks, the active-filled cream resulted in a 15.4% increase in elasticity, while the placebo cream showed a 10.7% response (*p* < 0.05) [26]. In another study involving saffron extract and avocado oil, scientists achieved similar statistically significant effects after 6 and 12 weeks of cream use [27]. In our study, the use of honey creams was not associated with an increased elasticity response after 4 weeks of use (*p* < 0.05). The slight response connected to time could be attributed to improvements after 15 min of initial use, possibly due to short-term stratum corneum moisturization.

Ambiguous results were obtained with the Visioscan VC 98 USB probe, which measures skin smoothness by calculating skin gray tones directly from live skin images. Taking into account the skin condition before and after four weeks of hand treatment, a statistically significant change in smoothness was observed only with the 5% (*w*/*w*) honey cream. This may be connected to the relatively small study groups. However, the observed phenomenon could be explained by the optimum ratio of water and humectant repeatedly delivered into the stratum corneum, as both too low and too high humectant contents were less efficient in smoothing the top layer of the skin. On the other hand, an average improvement level of 21.3% for the 5% *w*/*w* honey cream is rare. When considering commercial anti-wrinkle creams, samples collected by Portuguese scientists rarely demonstrated statistically significant effects on smoothness [28]. Moreover, creams containing the popular anti-wrinkle substance retinyl palmitate were found to improve facial skin smoothness by only 6.45% after 1 month [29].

Considering the unclear results obtained with the Visioscan VC 98 USB, the Visio-line VL 650–Quantridies was used to clarify the evidence of honey’s anti-wrinkle action. The difference between these two methods lies in calculations and skin imaging, as the Visioline VL 650 reads images obtained from skin replicas, which may reduce deviations from live imaging. Visioline is also a good method to measure precise wrinkle characteristics, such as mean wrinkle depth. There was a statistically significant reduction of around 10% in mean wrinkle depth for all the examined honey creams, with no significant effect observed for the placebo cream. These results confirm the contribution of honey in smoothing the skin surface after four weeks. Among bee products, the anti-wrinkle action has been confirmed for bee venom, which reduced facial mean wrinkle depth after 12 weeks with statistical significance [30]. Given the studies presented herein, honey’s position in commercial anti-wrinkle skincare products could be elevated. Further investigations are warranted.

## 4. Materials and Methods

### 4.1. Formulations Preparation

The composition of the O/W hand cream was experimentally determined to minimize the perception of stickiness upon skin application. The composition was devised in four different ratios: 0%, 5%, 10%, and 15% (*w*/*w*) honey content. The formulations were prepared in small batches using the hot-emulsification and homogenization process under controlled laboratory conditions in Przedsiębiorstwo Farmaceutyczne Farmapol Sp. z o. o. The formulation of the cosmetic products was in accordance with Regulation (EC) No 1223/2009/EC of the European Parliament and the Council of 30 November 2009 on cosmetic products with subsequent updates. The ingredients utilized in the cream vehicle were of cosmetic or higher-grade quality and included purified water (Przedsiębiorstwo Farmaceutyczne Farmapol, Poznań, Poland), Olive Oil (Alfa Sagittarius, Kraków, Poland), Caprylic/Capric Triglyceride (Masso, Warszawa, Poland), Cetearyl Alcohol (Standard, Lublin City, Poland), Stearic Acid (Standard, Lublin City, Poland), Symbiomuls GC (Adara, Warszawa, Poland), Kahlwax 8108 (Biesterfeld, Hamburg, Germany), Glycerin (Oqema, Korschenbroich, Germany), Vivastar CS 302 SV (JRS, Warszawa, Poland), Dub Diol (Brenntag, Kędzierzyn-Koźle, Poland), Symsave H (Aromat-Vertex, Kościan, Poland), Beta-Carotene (Jar Aromaty, Warszawa, Poland), and Parfum (Nordmann, Warsaw, Poland). Parfum and dye were added to effectively mask the scent and color of honey in adherence to this study’s single-blinded standards. 

The multifloral honey (Marian Sikora) was sourced from the Greater Poland region in Poland and complied with the PN-88/A-77626 Polish Standard and fulfills the Council Directive 2001/110/EC of 20 December 2001 relating to honey (12.1.2002 L10/47-52) and Revised Codex Alimentarius standard for honey 12-1981 standards and standard methods (Vol. 11). The collected batch of honey underwent a comprehensive examination according to specific parameters, yielding the following results: 17.6 (*w*/*w*) water content, 15.92 mg/kg hydroxymethylfurfural, 70.71 (*w*/*w*) reducing sugars (glucose and fructose), 26.19 diastatic number, 0.27 mS/cm electrical conductivity, and less than 0.5% (*w*/*w*) saccharose and melezitose (Supplementary S1).

In the cream variations containing 0–10% (*w*/*w*) honey, the gap in the content was replaced with purified water, adjusted to constitute 15% (*w*/*w*) of the formulation. The study samples were filled into pharmaceutical-grade aluminum 30 g tubes. These tubes were sealed and appropriately labeled to conform to the single-blinded study protocol. The stability of the cosmetic formulations and their compatibility with packaging were assessed before this study to ensure sample integrity throughout the application period.

### 4.2. Study Design

**Figure 11:**
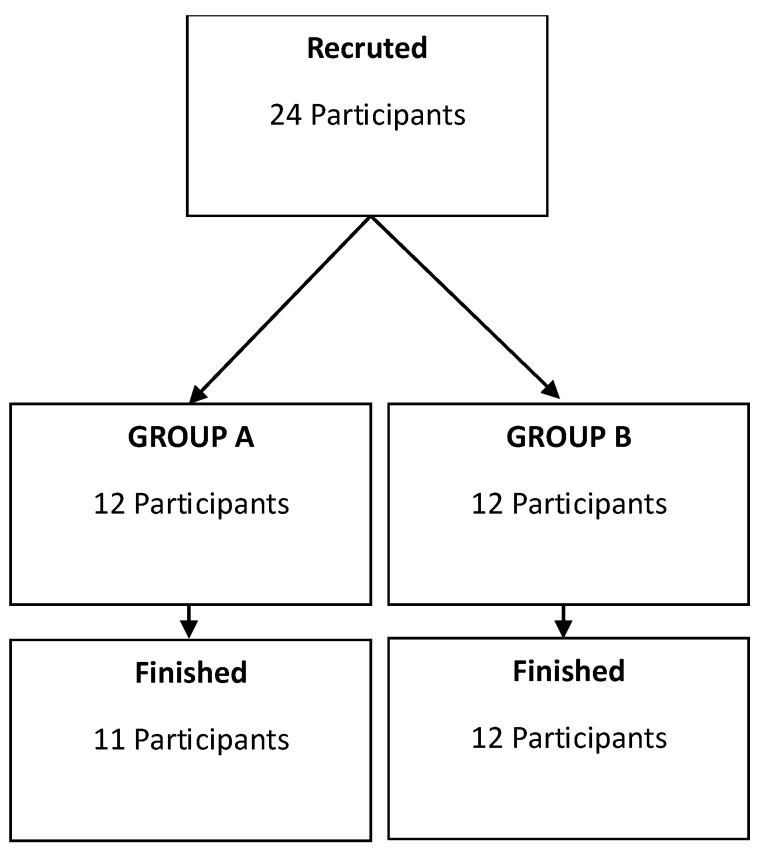
Division and progress of the present study. Hand creams with 0% and 10% (*w*/*w*) honey content were assigned to Group A, whereas hand creams with 5% and 15% (*w*/*w*) honey content were assigned to Group B.

The formulations underwent testing encompassing microbiology, preservation (challenge test), dermatology (patch test), stability, and compatibility with packaging to adhere to the safety standards mandated for cosmetic products. For this study, 24 participants from Poland (20 women and 4 men) aged 24 to 58 years were recruited. All participants confirmed a lack of past allergic reactions to honey or any other ingredient in the formulation. They were instructed not to use any other hand moisturizers at least one h before the tests. The participants were divided into two groups: Group A received a blinded pair of 0% and 10% (*w*/*w*) honey hand cream, while Group B received a blinded pair of 5% and 15% (*w*/*w*) honey hand cream (as shown in Figure 11). The age difference between the two groups was confirmed: t (11) = 0.549; *p* = 0.59, with means for Group A and Group B of 40.0 and 42.4, respectively. Sex distribution included 1 man and 11 women in Group A and 3 men and 9 women in Group B. Volunteers were directed to apply the cosmetics twice daily, respectively, on the left and right back of their hands. Throughout this study, using other hydrating hand care products was prohibited to eliminate confounding variables. The experiment encompassed three skin assessment time points: before cosmetics application, 15 min after the first application, and finally after four weeks of regular use. Before the apparatus examination, volunteers waited for 15 min to allow their skin to adjust to room conditions (40–45% humidity, 22–24 °C). Before the 4-week assessment, participants refrained from using the tested cosmetics for 1 h following the skin condition evaluation to mitigate the influence of short-term effects. Questionnaires were completed after four weeks of cosmetic use and following all the tests. This study was completed by 23 participants, with 12 in Group A and 11 in Group B.

### 4.3. Quantitative Methods

The participant questionnaire included rating questions regarding usage and sensory evaluation. The assessment utilized a scale ranging from 0 (very unfavorable) to 5 (very favorable), focusing on parameters like spreadability, absorption rate, and the sensation of a hydrating film on the skin. Formula stickiness was evaluated on a scale from 0 (no stickiness) to 10 (very sticky, honey-like residue). The purpose of this rating scale was to ascertain if the honey content influenced the sensory aspects of the cream, especially stickiness.

A non-invasive assessment of skin conditions was conducted by using the Courage&Khazaka Electronic GmbH (Köln, Germany) apparatus, equipped with probes to measure various skin parameters. These included moisture level, transepidermal water loss, elasticity, smoothness, wrinkle area, and depth. The measurement procedures were aligned with the apparatus manuals.

To measure skin hydration, the Corneometer CM 825 probe, which employs the capacitive method, was utilized. The hydration level of the skin is expressed in arbitrary units (a.u.) by measuring the dielectric constant of the stratum corneum. Higher a.u. values correspond to greater skin hydration. The probe was positioned on the middle back of the hands.

The Tewameter TM 300 probe assessed skin barrier function, specifically the parameter of transepidermal water loss expressed in g/m^2^/h. This cylindrical probe features two pairs of sensors to measure relative humidity above the stratum corneum. The parameter is calculated using Fick’s law of diffusion and the gradient of water density between the sensor pairs. The probe was placed on the middle back of the hand, and the average value was obtained after 20 s of sensor temperature adaptation. A lower parameter value indicates better skin barrier function.

The Cutometer dual MPA 580 probe measured skin elasticity. Employing a vacuum, the probe gently pulls adjacent skin. The degree of skin deformation and relaxation is monitored in real time, generating a traceable diagram. The R2 parameter, representing overall elasticity, was used in the statistical analysis. It ranges from 0 to 100%, with higher values indicating more elastic skin. The probe’s settings included a 2-s vacuum on-time, 2-s off-time, and a pressure of 450 mbar. The probe was positioned on the back of the hands between the thumb and forefinger.

The Visioscan VC 98 USB probe evaluated skin smoothness. This high-resolution camera with a UVA lamp captured images that were interpreted using grayscale distribution to calculate skin surface smoothness. Placed on the middle back of the hands, the probe’s SEsm (smoothness) parameter was employed in statistical analysis. SEsm is determined by the diversity of gray tones in the histogram; lower SEsm values indicate smoother skin surfaces.

Wrinkle surface area and depth were measured using the Visioline VL 650 – Quantridies apparatus. This method involves preparing a skin replica, obtained from the middle back of the hands, and placing it in the Visioline device. An illuminated light at a 35° angle and a high-resolution camera created an image, measuring and calculating the variations in skin irregularities. The mean wrinkle depth and the percentage of wrinkle area were parameters used in the statistical analysis.

### 4.4. Statistical Analysis

Statistical analysis was carried out to ascertain the significance of variances between groups and to elucidate the impact of honey content percentage on skin parameters. To this end, the analysis involved dependent Student *t*-tests and repeated-measures analysis of variance (ANOVA) with post hoc Bonferroni correction. To determine the differences between groups, a post hoc analysis with Bonferroni correction was performed. Additionally, dependent Student *t*-tests were employed to compare the means of the paired groups. Separate Student *t*-tests were conducted for each group to contrast the measured skin parameters between them. The baseline parameters for each skin measuring test and honey cream group were checked to determine if the differences were not statistically significant.

Additionally, the analysis incorporated an analysis of variance (ANOVA) to assess the influence of honey content on each skin parameter across multiple groups. The ANOVA methodology facilitated the concurrent comparison of average values across the groups, enabling the identification of any potential differences in outcomes between them. All statistical analyses were executed using IBM SPSS, version 28.0.1, with the significance level set at *p* < 0.05.

## 5. Conclusions

Honey, as a natural ingredient, offers numerous advantages, including biocompatibility, a relatively low cost, consumer popularity, and its status as a renewable resource, which aligns with the principles of sustainable development and beekeeping culture. However, it also comes with certain technological challenges, such as its thermolability, tendency to crystallize, and low sticky point temperature, which can result in a sticky sensation after product application. These challenges can be overcome through careful formulation processes and production technology planning.

Honey holds significant market potential, although its effectiveness, as demonstrated through instrumental methods data, remains somewhat limited. Our research sheds light on the impact of honey-infused hand creams on the skin, considering varying honey content levels and both short-term and long-term use. Our study affirmed the time and percentage-dependent moisturizing effect of honey creams on the skin, which was anticipated given prior scientific findings and the theoretical concept of honey’s humectant mode of action.

The promising results of our study include the anti-wrinkle and skin-smoothing effects of honey creams across all the tested percentage variations (≥5% *w*/*w*). The unexpectedly substantial improvements of 18.25% to 21.29% in wrinkle area and 10.5% to 11.7% in mean wrinkle depth reduction after four weeks of regular cream use position honey as a potentially significant rejuvenating agent, comparable to well-established substances like retinol or ascorbic acid.

However, our research revealed an unexpected finding in the TEWL parameter readings with the 15% honey cream, which improved the skin’s barrier function less favorably compared to the 5% and 10% honey creams. This could suggest that honey concentrations of 5–10% *w*/*w* might be more effective for enhancing the skin’s barrier function, while a 15% concentration could be optimal for providing better moisturization.

It is important to acknowledge potential limitations in our research, such as the small sample sizes in the statistical analysis or volunteers not fully adhering to the experiment’s requirements. Additionally, daily habits that could influence the outcomes, such as the number of daily hand washes, occupation, and the quality of hand care before the test, were not taken into account.

To conclude, further research and data collection on the use of honey in skincare are recommended. The unique properties of honey and its diverse impact on the skin warrant continuous exploration, which can lead to innovative cosmetic formulations and skincare products, as well as a better understanding of the mechanisms and effects of its action on the skin.

## Data Availability

Data is contained within the article and Supplementary Materials.

## Supplementary Materials

The following supporting information can be downloaded at: https://www.mdpi.com/article/10.3390/ph17121709/s1, Supplementary S1. Certificate of analysis of honey.
