# Supplementary material for: Innovative Honey-Based Product and Its Beneficial Effects Measured by Modern Biophysical and Imaging Skin Techniques
Source: Pharmaceuticals (Basel). 2024 Dec 18;17(12):1709. doi: 10.3390/ph17121709 (PMC11677624; doi:10.3390/ph17121709)
Supplement: Supplementary file 1 [file pharmaceuticals-17-01709-s001.zip › pharmaceuticals-3277419-supplementary.pdf]

Supplementary S1. Certificate of analysis of honey.

LABORATORIUM PRODUKTÓW PSZCZELICH
GOSPODARSTWA PASIECZNEGO „SĄDECKI BARTNIK" Sp.z o.o
33-331 STRÓŻE 235

A N A L I Z A 336/2021

Stróże 18.10.2021

Przedmiot Analizy: Próbka miodu dostarczona przez

Pasieka Sikora Mateusz Sikora ul. Poznańska 144 64-330 Opalenica

Wyniki analiz w stosunku do wymagań zgodnych z Rozporządzeniem Ministra Rolnictwa i Rozwoju Wsi z dnia 18 luty 2004 roku w sprawie szczegółowych wymagań w zakresie jakości handlowej miodu (Dz. U. Nr 40, poz. 370), z późniejszymi zmianami.

Oznaczone parametry	Numer próbki<br>01 z partii 900 kg data zbioru 24.06.2021	Norma
Zawartość wody (%)	17,6	Do 20
Kwasowość ogólna (ml 1 N NaOH/100g)		1-5
Przewodność właściwa(mS/cm)	0,27	N- nie więcej niż 0,8<br>S- nie mniej niż 0,8<br>NS – 0,6-0,8<br>SL – 0,81 – 0,95<br>SI – powyżej 0,95
HMF (mg/kg)	15,92	Do 40
Liczba diastazowa	26,19	Od 8,0
Zawartość sacharozy z melecytozą (%)	<0,5	Do 5
Zawartość cukrów redukujących (suma glukozy i fruktozy) (%)	70,71	nie mniej niż 60g/100 g w miodzie nektarowym 45g/100g w miodzie spadziowym i NS

Badania przeprowadzono wg RMRIRW z dnia 14.01.2009 z późniejszymi zmianami.

N – miody nektarowe
SL – miód ze spadzi liściastej
S- miody spadziowe
SI – miód ze spadzi iglastej
NS – miód nektarowo - spadziowy

Sporządził
SĄDECKI BARTNIK
Laboratorium Analityki
Produktów Pszczelich

Zatwierdził
Kierownik laboratorium
mgr inż. Alicja Cetnarowska
